# A Tri-fusion Reporter Mouse Reveals Tissue-Specific FGF1B Promoter Activity *in vivo*

**DOI:** 10.1038/s41598-019-47641-3

**Published:** 2019-07-31

**Authors:** Shan-Wen Liu, Ching-Han Hsu, Mei-Ru Chen, Ing-Ming Chiu, Kurt M. Lin

**Affiliations:** 1 Institute of Biomedical Engineering and Nanomedicine, National Health Research Institutes, Zhunan, Miaoli Taiwan; 2 Department of Biomedical Engineering and Environmental Science, National Tsing-Hua University, Hsinchu, Taiwan; 3 Institute of Cellular and System Medicine, National Health Research Institutes, Zhunan, Miaoli Taiwan; 4 Department of Biomedical Imaging and Radiological Sciences, National Yang-Ming University, Taipei, Taiwan

**Keywords:** Bioluminescence imaging, Spermatogenesis, Molecular imaging, Reporter genes

## Abstract

Transgenic mice harboring imaging reporters take full advantage of imaging technologies in studies using living mice. Here, we established a tri-fusion multimodal reporter gene containing fragments from firefly luciferase, enhanced green fluorescent protein, and herpes simplex virus type 1 thymidine kinase and generated tri-fusion reporter Tg mice. Fibroblast growth factor type 1 (FGF1), a multifunctional mitogen to a wide range of tissues, regulates proliferation of neural stem cells of the brain, where FGF1 expression is initiated through activation of the FGF1B (F1B) promoter. The reporter mouse under the control of the human F1B promoter enables visualization *in vivo* where F1B activity is elevated, including tissues not only in the brain but also in the nasopharynx, skull, spine, and testes, particularly in Leydig cells. Treating Tg mice with the alkylating agent busulfan, which is known to eradicate Leydig cells and disrupt spermatogenesis in mice, eliminated the reporter signals. Restoring Leydig cells recovered reporter expression, indicating that the reporter can be used as a surrogate marker for Leydig cells. The F1B tri-fusion reporter mouse model can be utilized in longitudinal monitoring of the health status of the male reproductive system, such as in studies exploring the toxicity of chemicals to spermatogenesis.

## Introduction

Reporter imaging utilizes the reporter products as surrogate markers to indirectly visualize complicated biological events or processes that are difficult to study otherwise. Multimodal reporter genes combine coding sequences for fluorescence proteins of various colors, luciferases, and nuclear medical imaging reporters, which are placed in *cis* or as a single fusion protein, have been utilized widely in imaging studies. The main advantages of multi-modality imaging are to utilize combined strengths of multiple imaging platforms while gaining flexibility, particularly for longitudinal tracing and measurement on same subjects^1–6^.

Fibroblast growth factor 1 (FGF1), also known as acidic fibroblast growth factor, is the universal FGF that can activate all FGF receptors. FGF1 activates signal transduction cascades for a broad range of biological processes, including embryonic development, cell growth, morphogenesis and remodeling^7–10^. FGF1 regulates lineage- or tissue-specific processes, for example, activating angiogenesis of endothelial cells^9,11^, sustaining growth and self-renewal capacity of neuroectoderm-derived cells such as neural stem cells^12–14^, and modulating the differentiation of mesoderm lineage tissues^15–17^. Alternative promoters direct the tissue-specific expression of FGF1. Four 5′ upstream untranslated exons (1A, 1B, 1C, and 1D) in the human FGF1 promoter due to distinct transcriptional start sites, aka alternative transcription initiation, have been identified^18–20^. F1B is the major transcript expressed in the brain and retina^21^, while the other transcripts, F1A, F1C, and F1D, are the dominant transcripts in the kidney^19^, vascular smooth muscle cells, and fibroblasts, respectively^22^. F1B mRNA is expressed at the sensory and motor nuclei of the brain stem and spinal cord^23^. Expressing SV40 T antigen in transgenic mice under the control of the human F1B promoter (−540 to +31 bp) resulted in a high incidence of tumors at the olfactory bulb, ventral forebrain, subventricular zone, thalamus, striatum, and tegmental area where neural stem/progenitor cells are known to be abundant^24,25^. By using an F1B promoter-driven GFP vector and in Tg mice, GFP-positive neural stem/progenitor cells can be readily isolated from the brains of developing or adult mice^12,26,27^. However, limited by the penetrating depth and signal intensity of GFP, F1B-GFP mice were unable to demonstrate whole body F1B promoter activity *in vivo*.

In this study, we generated a trimodal imaging reporter (abbreviated TMIR) Tg mouse expressing a tri-fusion gene consisting of (N terminus → C terminus) firefly luciferase, enhanced green fluorescent protein (eGFP), and HSV1Δ*tk* under the control of the human F1B promoter (shortened as F1B-TMIR). Compared to F1B-GFP mice, F1B-TMIR mice take advantage of *in vivo* imaging using bioluminescence, PET, and SPECT and enable the discovery of F1B promoter activity in male testes. Furthermore, we demonstrated that F1B-TMIR mice can be used to reveal the disruption of spermatogenesis in male mice.

## Results

### Vector construction and cell culture study

**Figure 1:**
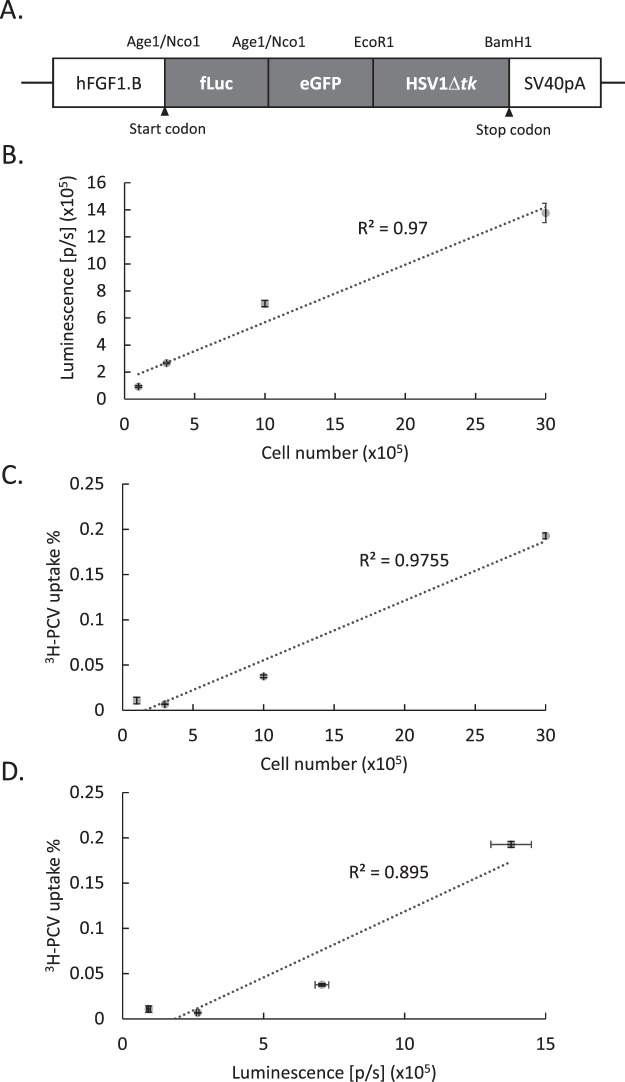
Vector construction and assays for luciferase activity and ^3^H-PCV uptake in CHO-k1 cells. (**A**) Structure of the F1B-TMIR transgenic vector. (**B**) Measured luciferase activities in increasing numbers of F1B-TMIR-transfected CHO-k1 cells. p/s = photons per second. (**C**) The percentage of ^3^H-PCV uptake in 4 h by different numbers of F1B-TMIR-transfected CHO-k1 cells. First, 0.5 μCi/ml of ^3^H-PCV was added to cells, and the percentage of uptake is defined in the Methods section. (**D**) Correlation of the percentage of ^3^H-PCV uptake and measured luciferase activity.

Tri-fusion reporter vectors containing coding sequences of firefly luciferase, eGFP, and HSV1Δ*tk* genes under the control of the CMV or F1B promoter were constructed as described in the Methods section (Fig. 1A). The order of reporter elements in TMIR is similar to the tri-fusion reporter described previously by Ray *et al*.^6^. We validated the functions of each component in the fusion reporter in CHO-K1 cells. The green fluorescence of TMIR was located in both the cytoplasm and nucleus, compared to the primarily nucleus-localized fluorescence by the eGFP-*tk* vector in which eGFP was fused to full-length HSV*-tk*^28^, indicating that the removal of the N-terminal residue of HSV1*tk* in TMIR caused increased cytoplasmic localization (Fig. S1). The enzymatic activity of luciferase and HSV1 thymidine kinase in the TMIR fusion protein was measured by luciferase assays and by measuring the uptake of an HSV*tk* substrate, tritium-labeled penciclovir ([^3^H]PCV)^29,30^, in F1B-TMIR vector-transfected CHO cells (Fig. 1B,C). Figure 1B,C demonstrated that luminescent light output and [^3^H]PCV uptake strongly correlated with cell numbers with R^2^ > 0.97. The correlation of luminescence to [^3^H]PCV uptake was strong with R^2^ > 0.89 (Fig. 1D). HSV1Δ*tk* activity in TMIR was also indirectly measured by the sensitivity to ganciclovir (GCV), which induces cytotoxicity in cells expressing HSV1tk (Fig. S2). The measured luciferase activity of TMIR was used as a reading of cell viability and was thus reduced by GCV. Compared to the TMIR vector driven by CMV, the F1B-TMIR vector resulted in significantly lower luminescence, indicating a low basal activity associated with the F1B promoter. GCV treatment reduced the light output of both vectors, reflecting the induction of cell death. The luminescence by CMV-TMIR was more sensitive to added GCV than F1B-TMIR, presumably due to a higher TMIR expression (Fig. S2).

### F1B-TMIR Tg mouse generation and optical imaging

**Figure 2:**
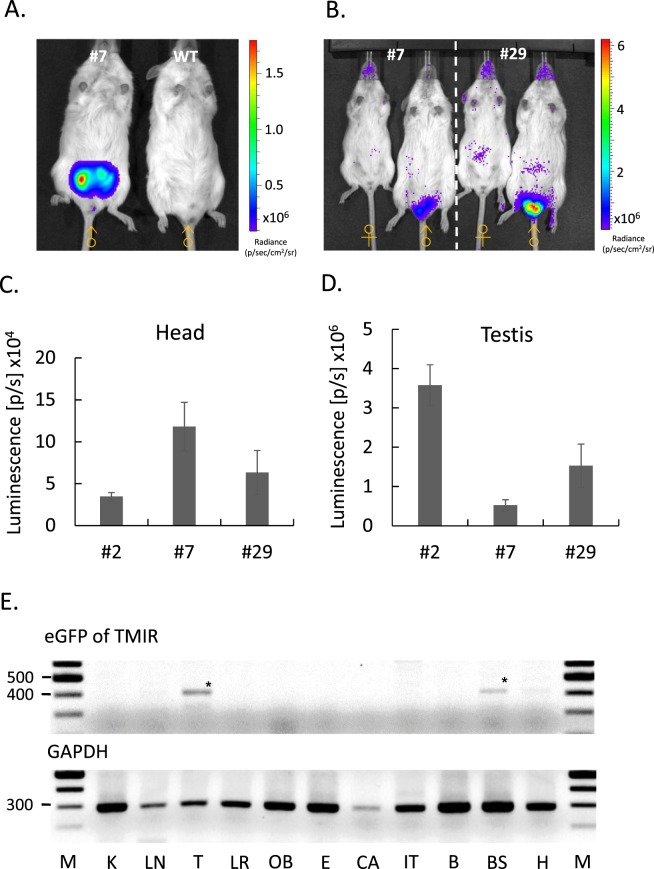
*In vivo* optical imaging for F1B-TMIR Tg mice. (**A**) Bioluminescence imaging of a male F1B-TMIR mouse from line #7 and a male WT mouse. (**B**) Imaging results for both genders of F1B-TMIR mouse lines #7 and #29, showing strong signals in the testes of both lines. (**C**,**D**) Different Tg lines all displayed strong bioluminescence of the testis, quantitated from imaging results of Tg mouse lines #2, 7, and 29. The ROI in (**C**) enclosed the head region (N = 3–11 in each line), and the signals in (**D**) were proven to come from testes in the ROI enclosed in the abdominal region (N = 4–6 in each line). (**E**) Representative RT-PCR results showing TMIR expression in various organs. 405 bp eGFP fragment of TMIR (*) and 290 bp GAPDH fragment is shown. Kidney (K), lung (LN), testis (T), liver (LR), olfactory bulb (OB), eyes (E), cartilage (CA), intestines (IT), forebrain (B), brain stem (BS), and heart (H).

**Figure 3:**
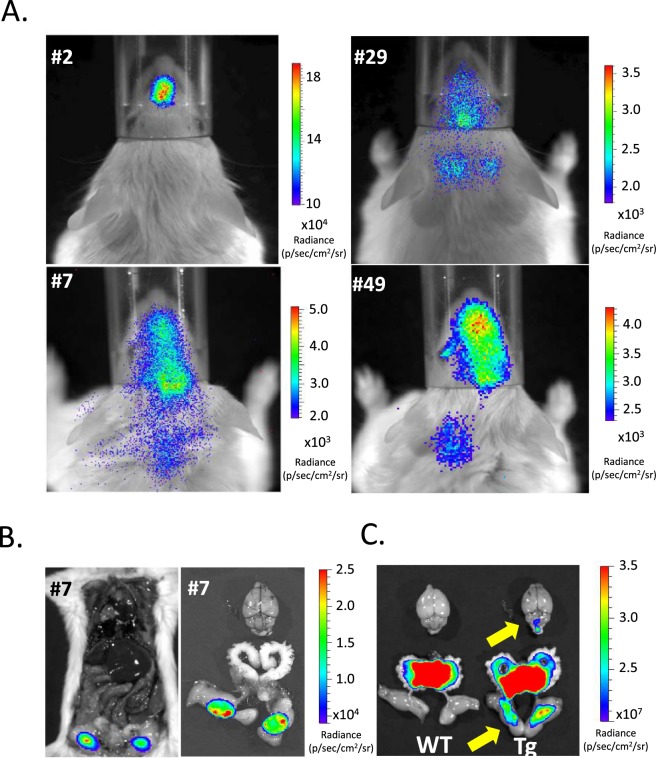
Bioluminescence imaging of F1B-TMIR Tg mice. (**A**) Patterns of imaging signal distribution in the head region of lines #2, 7, 29, and 49. (**B**) Bioluminescence images of a male line #7 mouse. Left, after given luciferin through i.p for 5 min, the mouse was imaged with the chest and abdomen opened. Right, images of brain and reproductive organs of the same mouse. (**C**) Green fluorescent images of brain and reproductive organs of a male wild-type (WT) mouse and Tg line #7. Strong autofluorescence in the vas deferens, prostate glands, and seminal vesicles was also present in WT mice and was not due to TMIR. A green fluorescence signal in Tg mice was found at the testis and brain stem (yellow arrows).

We used the F1B-TMIR vector to generate multiple FVB Tg mouse lines. Tail DNA Southern blots of various Tg lines are shown in Fig. S3, and four lines, #2, 7, 29, and 49, were studied. Bioluminescence imaging of the mice and RT-PCR revealed that TMIR was expressed in the head, including the olfactory bulb, eyes, and brain stem (Figs 2A,B and 3). Strong bioluminescent signals were observed in the testes of male mice of all Tg lines (Figs 2A,B and 3B), as shown in Fig. 2C,D. The testis signals were 5 to 100 times the head signals. After completing the live imaging, we sacrificed the mice and performed *ex vivo* imaging immediately to validate that bioluminescence was sourced from the testes (Fig. 3B). Although high tissue autofluorescence was observed, green fluorescence was observed above the tissue background fluorescence in the testes and brain stem (Fig. 3C). A RT-PCR-based assay was used to validate transgene expression in various organs. The results showed that the eGFP part of the TMIR transcripts was detected in the testis and brain stem (Fig. 2E). Furthermore, we analyzed TMIR expression in various organs by quantitative PCR (Fig. S6), and the gene expression results agree with the imaging results.

### HSV1Δ*tk* nuclear imaging of F1B-TMIR Tg mice

**Figure 4:**
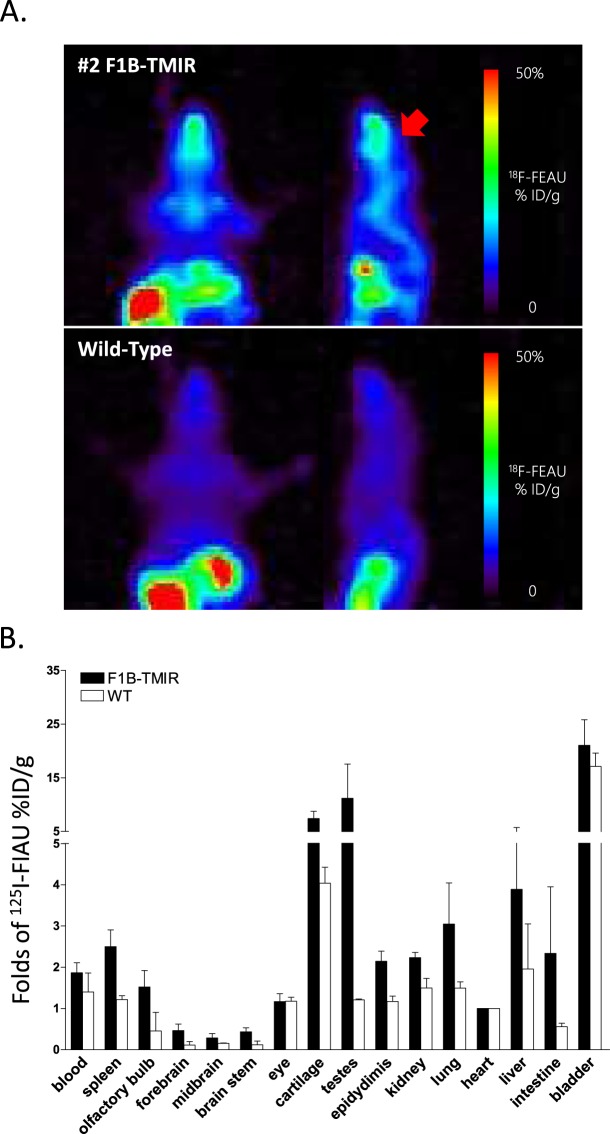
HSV1Δ*tk* mPET imaging and [^125^I] FIAU biodistribution. (**A**) [^18^F] FEAU mPET images in coronal and sagittal views of a male Tg line #2 (Top) and a WT mouse (Bottom). Uptakes of tracers in Tg mouse head, including the turbinate, cranial skull, and spinal cord. The brain is considered a low-count region (arrow). (**B**) Comparison of 24 h [^125^I] FIAU uptake in various organs by Tg line #2 and WT mice. Organs were collected and weighed, and the contained activity was measured by a γ-counter. The uptake in the heart of each animal was used to normalize the uptake by other organs.

HSV1Δ*tk*-specific microPET imaging by using [^18^F]FEAU was utilized for the distribution and expression level of TMIR. Unlike the luciferase substrate luciferin, which is a small molecule able to cross the intact blood brain barrier (BBB), the efficiency of FEAU to cross the intact BBB was uncertain. We opened the BBB according to a previously described method by injecting 25% D-mannitol through the tail vein^31,32^, followed by an immediate injection of [^18^F]FEAU (1.11 MBq). A PET scan was performed 2 hours after probe injection. As shown in Fig. 4A, uptake of [^18^F]FEAU was noted at the turbinate, spinal cord, and possibly at the olfactory bulb and skull of F1B-TMIR mice. There was no significant uptake at the above regions in WT animals. The F1B-TMIR mouse brain was not significantly labeled by [^18^F]FEAU (arrow in Fig. 4A), suggesting that even when assisted by mannitol injection, [^18^F]FEAU did not effectively cross the BBB. Strong uptake was observed in the abdominal region of male mice, but the fast decay of [^18^F]-labeled probes requires animals to be imaged within a few hours of probe injection; thus, [^18^F]FEAU retained specifically in testis or to be excreted by urine was not distinctly distinguished. A SPECT/CT scan with [^125^I]FIAU was used to image animals at 2 hours after probe injection. The results of SPECT/CT using [^125^I]FIAU were in agreement with the [^18^F]FEAU PET results showing uptakes at the olfactory bulb and turbinate, brain stem, and testes (Fig. S4A). Uptakes of [^125^I]FIAU by various organs were measured quantitatively and are represented by the folds of uptake by the F1B-TMIR mouse organ normalized to the uptake in heart and compared to WT organs (Fig. 4B). All organs of F1B-TMIR mice had higher uptakes for [^125^I]FIAU than WT mice. Testes and brain stem had the greatest increase in uptake in F1B-TMIR mice, presumably due to high TMIR expression in these two organs. We used [^131^I]FIAU imaging to confirm the uptake of the TK probe by testes in addition to the uptake in the heads because of the absence of similar signals in female mice (Fig. S4B).

### Reporter expression in F1B-TMIR mouse brains

**Figure 5:**
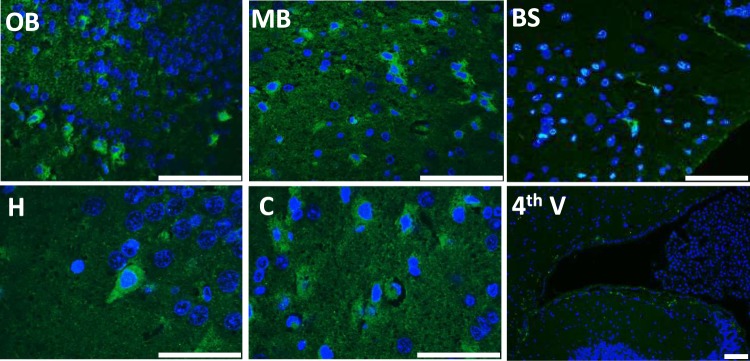
Luciferase expression in F1B-TMIR mouse brain. (**A**) TMIR in mouse brain sections was detected by anti-luciferase antibody. Scale bar = 50 μm. OB: olfactory bulbs; MB: midbrain; BS: brain stem; H: hippocampus; C: cortex; 4^th^V: the fourth ventricle.

TMIR expression in the mouse brain *in situ* was detected by immunofluorescence (IF) with anti-luciferase antibodies on paraffin-embedded brain sections. As shown in Fig. 5, luciferase expression was detected in the olfactory epithelium, olfactory nerves, midbrain, white matter of the cerebellum, and ventricular system of the F1B-TMIR mouse brain. Most of these regions were in agreement with previously reported regions of high tumor incidence in the F1B-Tag mouse^24^. The pattern of TMIR expression in the brains of various Tg lines is summarized in Table 1.

**Table 1:** Table of reporter expression patterns in brain.

	Olfactory bulb	Striatum	Thalamus	Mid-brain	Hippocampus	Cortex	Brain stem	Ventricular system			
								Lat.	Aq.	3rd	4th
#2	++	−	−	+	−	−	++	+	++	+	++
#7	++	−	−	+	−	−	++	++	++	+	++
#29	++	−	−	−	+	+	++	++	++	++	++

++strong expression; +expression; −expression undetected.

### Leydig cells in the testis of F1B-TMIR mice express imaging reporters

**Figure 6:**
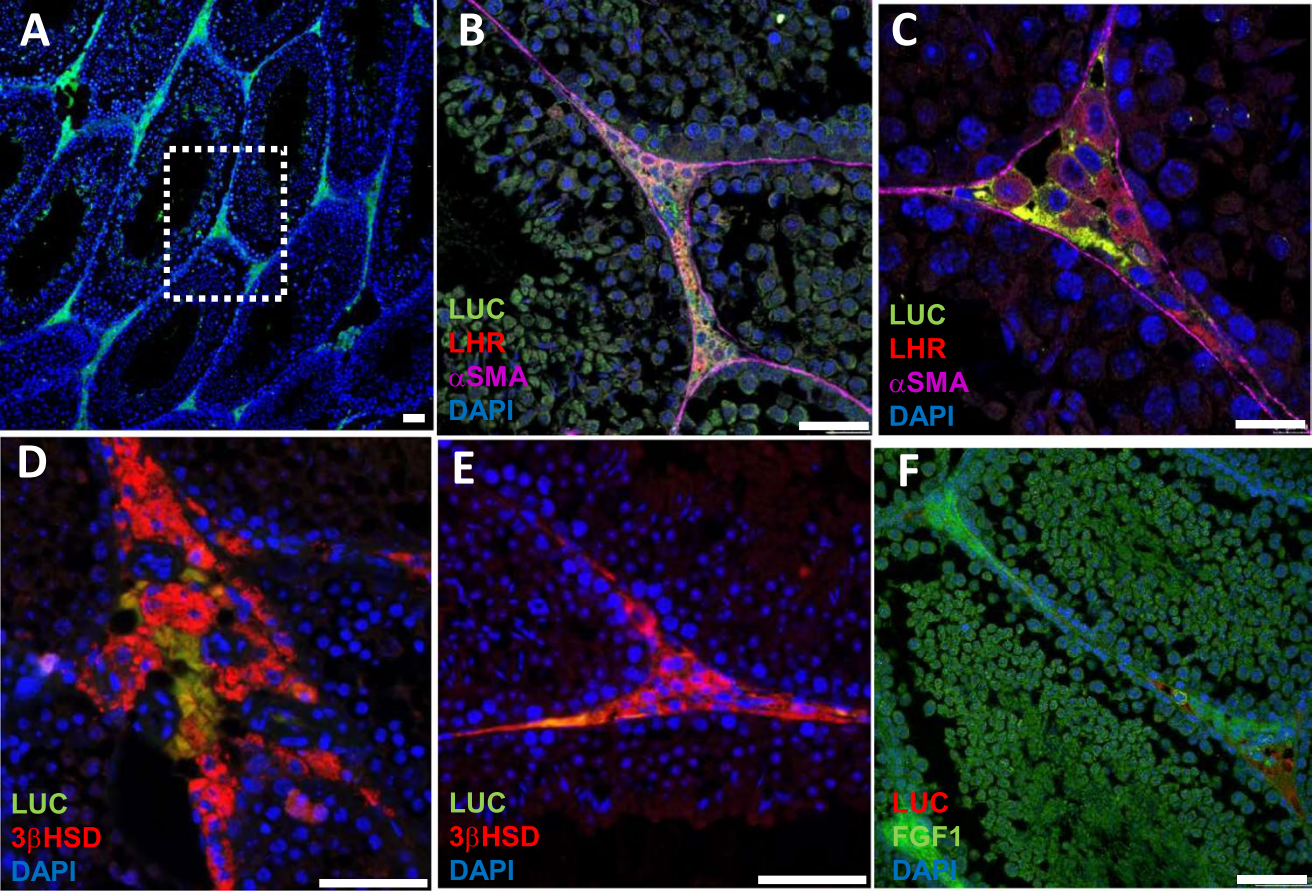
Luciferase expression in F1B-TMIR mouse testes. (**A**) Luciferase expression primarily in Leydig cells between seminiferous tubules of F1B-TMIR mouse mice. (**B**–**E**) Luciferase expressed in interstitial Leydig niches, including Leydig cells and vascular cells. Anti-LHR (**B**,**C**) and anti-3β-HSD antibodies (**D**,**E**) are used as markers for Leydig cells. Anti-α smooth muscle actin (α-SMA) antibody was used to delineate the surface mesenchyme of seminiferous tubules. (**F**) Expression of luciferase and FGF1 colocalizes at interstitial regions of the testis. Scale bar = 50 μm.

To study testis TMIR expression in detail, we performed IF detection on sections of Tg mouse testes. Cells with TMIR expression were primarily located at the interstitial region between seminiferous tubules (dashed line in Fig. 6A) and confirmed as Leydig and vascular cells by costaining with anti-luteinizing hormone receptor (LHR) and anti-3β-hydroxysteroid dehydrogenase (3β-HSD) antibodies, both of which are considered markers of Leydig cells (Fig. 6B–E). To study the correlation of TMIR and FGF1 expression in the testis, anti-FGF1 and anti-luciferase antibodies were used, and colocalization of TMIR and FGF1 at the interstitial region of the testis was found (Fig. 6F).

### F1B-TMIR as an *in vivo* reporter for spermatogenesis

**Figure 7:**
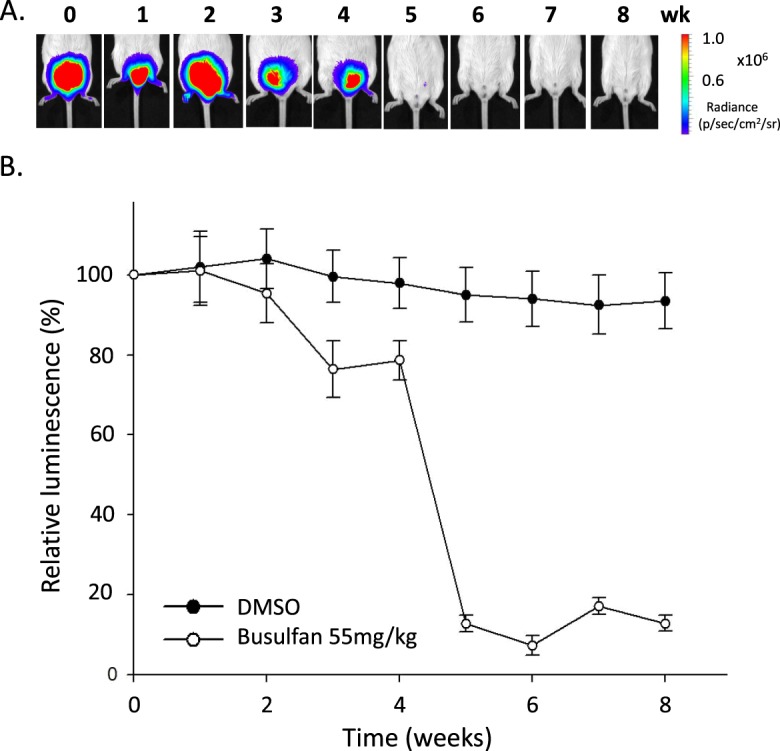
Longitudinal imaging of F1B-TMIR mice after a one-time high dose busulfan injection. (**A**) F1B-TMIR Tg mice treated by one-time 55 mg/kg busulfan injection followed by weekly bioluminescence imaging for 8 weeks. Time series images of the same mouse are shown. (**B**) Longitudinal changes in testis luminescence in mice treated with busulfan or DMSO. The luminescence was normalized to the value before busulfan or DMSO injection (week 0).

**Figure 8:**
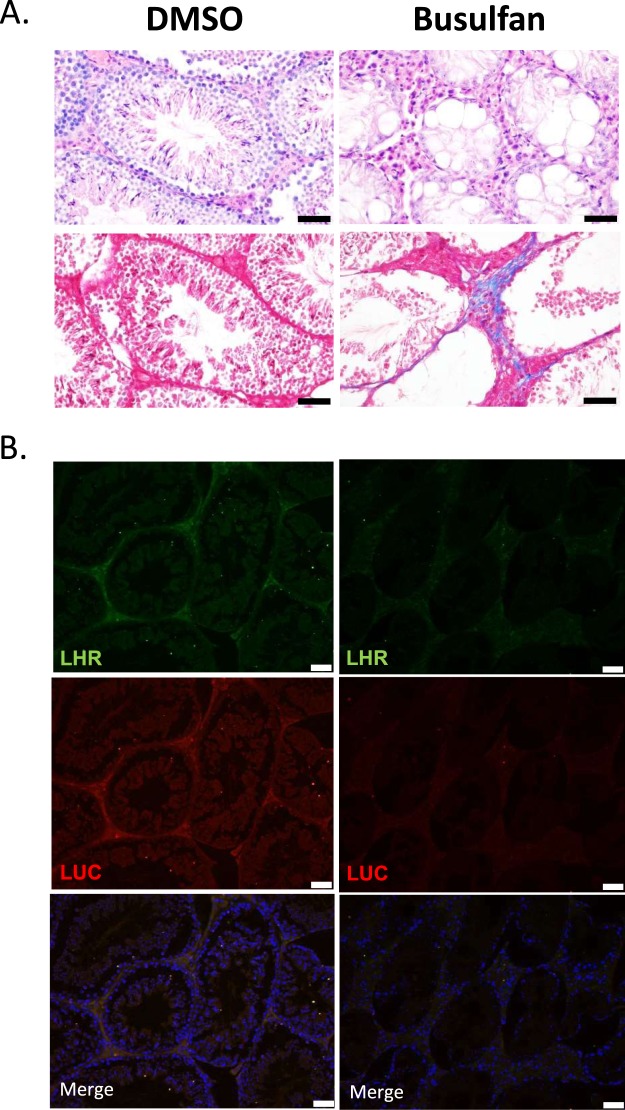
Pathology of F1B-TMIR mouse testis after busulfan injection. (**A**) Representative H&E staining (top) and Masson trichrome staining for fibrosis (bottom) of testes of DMSO-treated, or busulfan-treated Tg mice. (**B**) Leydig cell marker (LHR) and luciferase expression in the testis. Scale bar = 50 μm.

**Figure 9:**
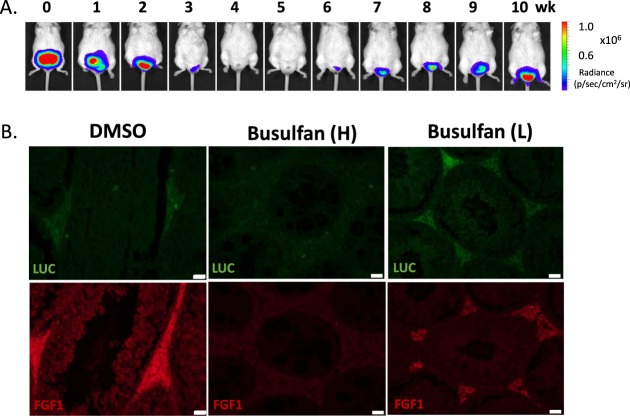
Bioluminescence imaging of F1B-TMIR mice after low-dose busulfan injection. (**A**) Time series images of the same mouse after a one-time 15 mg/kg busulfan injection followed by weekly bioluminescence imaging for 10 weeks. (**B**) Top. Luciferase expression in the testes of DMSO-, high-dose busulfan-, or low-dose busulfan-treated mice. Bottom. FGF1 expression in the corresponding sections. Scale bar = 20 μm.

After confirming testis TMIR expression in Leydig cells, we further studied whether TMIR expression can be used as a reporter in mice with dysfunctional Leydig cells. We investigated TMIR expression in mice with disrupted spermatogenesis that is regulated by hormones secreted by Leydig cells. Male F1B-TMIR mice were treated by one-time busulfan injection (55 mg/kg), an alkylating agent inducing Leydig cell apoptosis, and as a result, we observed a gradual reduction and total loss of TMIR signals (Fig. 7A). The largest decline in TMIR signals occurred in mice 4–5 weeks after busulfan injection (Fig. 7B). A disruption of testis structure and loss of cells by busulfan in interstitial and seminiferous tubules was observed (Fig. 8A). After 8 weeks, seminiferous tubules remained acellular without spermatogonia or Sertoli cells. The intertubule space originally occupied by Leydig cells was replenished by cells that did not express LHR (Fig. 8B) as a result of fibrocyte proliferation and interstitial fibrosis (Masson trichrome staining in Fig. 8A). The loss of Leydig cells and TMIR expression resulted from busulfan treatment (Fig. 8B). Furthermore, we explored whether mice with temporary Leydig cell death and recovery of spermatogenesis can be detected by changes in TMIR expression. Low-dose busulfan (15 mg/kg) injection is known to result in reversible germ cell depletion. After recovery from a low-dose busulfan treatment, TMIR expression in the testis was restored to some extent from a temporary complete loss of signals (Fig. 9A). Pathology of the testis revealed various degrees of recovery in the seminiferous tubules. While structures of some tubules appeared normal with sperm, spermatids, Sertoli cells, spermatogonia, and spermatocytes, other tubules displayed a disrupted structure, and some tubules did not contain any supporting cells. Widened spacing between tubules contained fibroblasts and loosely arranged Leydig cells (Fig. S9). Furthermore, we investigated whether testis TMIR levels correlated with FGF1 expression in these animal models and found that the number of FGF1-expressing cells was greatly reduced by high-dose busulfan. In low-dose busulfan-treated mice, Leydig cells regained luciferase and FGF1 expression in mice with recovered bioluminescence and resumed spermatogenesis (Fig. 9B).

## Discussion

Here, we report a tri-fusion reporter mouse in which the human F1B promoter drives a transgene containing luciferase, eGFP, and HSV1Δ*tk*. Imaging results revealed TMIR expression specifically in testes in addition to brain, skull, and spine. Strong testis TMIR expression led to the novel discovery of FGF1 expression in Leydig cells of testes. In the past, studying tissue- and stage-specific changes of promoter activity was difficult and was only available by using cultured cells or isolated organs. Promoter-driven reporter mice, such as F1B-TMIR mice, can visualize reporter expression *in vivo* and enable the novel discovery of specific promoter activation *in vivo* in unexpected tissues.

A tri-fusion reporter mouse model driven by a ubiquitous actin promoter has been described previously^30,33^. Compared to reporter mice in which reporters are often driven by a strong promoter or to models involving transplanted cells expressing reporters in large amount, imaging for F1B-TMIR mice is challenging because the Tg reporter is driven by an endogenous weak promoter. The fusion of multiple reporters in TMIR aided the flexibility of choosing suitable imaging methods in research. The results of bioluminescence and nuclear imaging demonstrated that these two modalities are more suitable for whole animal imaging than fluorescence imaging of detecting GFP. Nevertheless, the inclusion of GFP in TMIR will be useful for studies at the cellular level, such as fluorescence microscopy and flow cytometry. For example, selecting GFP (+) cells of F1B-GFP mice by fluorescence-activated cell sorting conferred the selected neural sphere/progenitor cells with enhanced proliferation and differentiation compared to cells not subjected to selection^12,26^.

FGF1 is expressed in a tissue-specific manner in adult animals. As such, F1A is expressed in the heart, kidney and adipocytes^19,34,35^, and F1B is expressed in the brain^20,24^ and testis (current study). However, the expression of FGF1 during development (from embryos to pups) is less well studied. There is only one report^36^, using the mouse embryonic cell lines, showing that F1B is activated during cardiac differentiation from ESCs. The technical platform described in this report should become an efficient tool in studying reporter gene expression in both spatial and temporal manners. This TMIR platform allows efficient scanning of the whole body and shows that the F1B signals are sequestered in the testis and brain. F1B activation in neural stem cells was originally one of the motivations to generate TMIR mice driven by the F1B promoter. In this study, low uptake for HSV1Δ*tk* probes in the brain is likely due to low TMIR expression in the brain as well as due to inefficient crossing of the BBB by all Δ*tk* probes tested, including FIAU and FEAU. Quantitatively addressing imaging probe transport across intact BBB remains a challenging task. According to a previously reported method^31,32^, 25% D-mannitol injection can partially open the BBB in rodents, but the locations and the homogeneity of BBB opening by mannitol were only roughly demonstrated. As shown in Fig. S7, mannitol followed by trypan blue injections resulted in an appreciable but rather limited increase of trypan blue distribution in the brain. Variations in imaging probe distribution at different brain regions would limit and simultaneously complicate the interpretation of imaging results for addressing gene expression patterns in the brain.

Located in the highly vascularized interstitial stroma between seminiferous tubules, Leydig cells synthesize and secrete testosterone by responding to the action of pituitary luteinizing hormone (LH)^37,38^. Hormones produced by Leydig cells support the development and regeneration cycles of male sperm. Although Leydig cells exhibit significant neuroendocrine characteristics, the origin of Leydig cells is still under debate. Vascular smooth muscle cells and pericytes have been proposed to be the progenitors of Leydig cells^38^. Imaging results of F1B-TMIR mice led to the unexpected finding of F1B promoter activity as well as FGF1 expression in Leydig cells. These findings in Leydig cells are consistent with the initial identification of the F1B promoter specifically in neural tissues. The molecular basis of the F1B promoter activity, e.g., which set of transcription factors interacting with the F1B promoter leads to the production of FGF1 and TMIR transcripts in Leydig cells, however, remains unidentified.

Leydig cells of F1B-TMIR mice were specifically labeled by tri-reporters and can be applied in studies for the mechanisms contributing to the formation, development, and regeneration of fetal and adult Leydig cells^39^. For example, we observed temporal changes in testis TMIR expression starting from 2 weeks old to 8 weeks old throughout the stages of development and maturation for male reproductive organs in mice (Fig. S8). Therefore, we believe F1B-TMIR mice are useful to studies requiring noninvasive and longitudinal monitoring of the health status of the male reproductive system, such as in exploring the toxicity to male spermatogenesis by environmentally toxic substances. In this regard, temporal changes in TMIR expression, reflecting interrupted and resumed spermatogenesis in mice treated with low-dose busulfan, prove the functions of F1B-TMIR mice.

In summary, our results demonstrated that (1) the tri-fusion reporter has the advantages of flexibility in choosing imaging platforms, (2) TMIR expression in F1B-TMIR mice correlates well with endogenous F1B promoter activity in various organs, (3) a high level of F1B promoter activity in Leydig cells suggests that FGF1 may be involved in pathways regulating testosterone hormone production, and (4) TMIR expression in Leydig cells provides a unique and useful feature of the F1B-TMIR mouse as a Leydig cell reporter mouse in studies relating to male reproduction.

## Methods

### Vector construction

The tri-reporter was constructed by linking the coding sequences of firefly luciferase, eGFP, and HSV1Δ*tk*, which has a truncation at the HSV1*tk* N-terminus. As previously described^28^, the HSV1*tk* (GenBank: CAA23742) coding sequence (a.a. 1–376) was inserted into the C-terminus of an eGFP expression vector (EGFP-C2, Clontech) at the EcoR1/BamH1 sites. The first 135 bp of HSV1*tk* in the eGFP-*tk* vector was removed by PCR-directed mutagenesis, resulting in the eGFP-Δ*tk* vector. The F1B promoter, which was isolated from an F1B-GFP vector^12,26^ as a 540-bp ApaL1/Age1 fragment, was used to replace the CMV promoter in the eGFP-Δ*tk* vector, resulting in the F1B-eGFP-Δ*tk* vector. The coding sequence of firefly luciferase (Luc) was isolated from the pGL2 vector (Promega) by PCR and inserted into the F1B-eGFP-Δ*tk* vector at the Age1 site to generate the F1B-Luc-eGFP-Δ*tk* vector (F1B-TMIR). Luc was similarly inserted into the eGFP-Δ*tk* vector to generate the CMV-TMIR vector. All vectors were verified by DNA sequencing.

### Fluorescence microscopy

eGFP expression in cells transfected with F1B-TMIR or CMV-TMIR was observed using an Olympus IX71 fluorescence microscope with Ex 490 nm/Em 520 nm filters. Immunofluorescence detection was performed on PTFE sections, and some immunofluorescent results were scanned by Leica TCS SP5 II confocal microscopy or Olympus confocal FV10i microscopy.

### [^3^H]penciclovir uptake assay

Validation of HSV1-Δ*tk* uptake in F1B-TMIR-transfected CHO-k1 cells was conducted as previously described with minor modifications^29^. After 48 h recovery from transfection, cells were aliquoted to varied numbers and incubated for 4 h with 0.5 μCi/ml of [^3^H]penciclovir (PCV). After incubation, the cells were washed three times with saline followed by cell lysis. Culture medium, wash saline, and cell lysates were collected, and the contained radioactivity was counted by a liquid scintillation counter (HIDEX 300 SL). A group of samples containing no cells in incubation was used in calculations as the total amount of ^3^H-PCV in reactions because the uptake in 4 h in cell lysate was typically less than 0.2% of the ^3^H-PCV added to incubation. A group of incubations with cells that did not express TMIR was used to measure the nonspecific uptake by CHO-k1 cells, with the value typically less than 20% of uptake by TMIR-expressing cells and was subtracted in calculations. The percentage of ^3^H-PCV uptake is defined as ([DPM]_TMIR Cell_) − [DPM]_Non-Specific_)/[DPM]_total_. Before the step of adding isotopes, separated cell aliquots were measured for luciferase activity.

### Transgenic mouse generation

All animal experiments were conducted in accordance with accepted standards of animal care and were approved by the Institutional Animal Care and Use Committee of the National Health Research Institutes, Taiwan. FVB/NarL transgenic mice were generated at Level Biotech, Inc. (Taipei, Taiwan) using an ApaL1/PvuII-digested F1B-TMIR vector. Six transgenic founders were generated, and transgenic lines from four of the founders (#2, #7, #29, and #49) were analyzed. The genotyping of transgene-positive mice was performed by PCR using the primers TTGACCGGTCACCATGGAAGACGCCAAA and CGTCGCCGTCCAGCTCGACCAG to amplify the transgene. Southern blotting of transgenic mice was performed on mouse tail DNA digested with NcoI, followed by 0.8% agarose electrophoresis and transfer to a nylon membrane. Full-length eGFP cDNA was labeled by [^32^P]αdCTP and used as a probe to detect inserted transgenes as a 3.6-kb band or a 5.2-kb band in the blot representing a tail-to-head or tail-to-tail arrangement of transgenes (Fig. S3).

### Bioluminescence imaging

Bioluminescence imaging was performed by using an IVIS Imaging system 200 or a Spectrum series (Caliper Life Sciences). The animal was anesthetized with isoflurane followed by intraperitoneal injection of D-luciferin (150 mg/kg; Caliper). The images were acquired 5 min after injection of the substrate and were repeated until the signal was attenuated or after 30 min.

### [^18^F]FEAU preparation and microPET imaging

[^18^F]2′-fluoro-2′-deoxy–1-β-D-arabinofuranosyl-5-ethyluracil) ([^18^F]FEAU) was synthesized as previously described^40–42^ by coupling 2′-deoxy-2′-[^18^F]fluoro-3, 5-di-*O*-benzoyl-α-D-arabinofuranosyl bromide with freshly prepared 2, 4-bis-*O*-(trimethylsilyl)-5-ethyluracil. Under gas anesthesia with isoflurane, the mouse was injected with 25% D-mannitol (120 μl) through the tail vein and then immediately injected with [^18^F]FEAU (1.11 MBq). microPET R4 (Concorde Microsystems) imaging was performed 2 h after the probe injection, and the sinogram data were acquired for 15 min. The sinograms were reconstructed by a fully 3D Bayesian reconstruction algorithm to improve quantitation accuracy^43^. The algorithm incorporated (1) the system normalization file that compensates for the detection efficiency of all detectors and (2) a smoothing image prior model that ensures the uniformity of spatial resolution^44^. The dimensions of the reconstructed images were 128 × 128 × 63, with voxel sizes of 1.0 × 1.0 × 1.21 mm^3^. The unfiltered image results were analyzed using the AMIDE package^45^.

### [^131/125^I]FIAU preparation, γ-camera and SPECT/CT imaging

Carrier-free [^131/125^I] (2′-fluoro-2′-deoxy-1-β-D-arabinofuranosyl-5-iodouracil) ([^131/125^I]FIAU) was synthesized from 5-trimethylstannyl-1-(2′-deoxy-2′-fluoro-1-β-D-arabinofuranosyl)-uracil (FTAU) by an oxidative iododestannylation method as previously described^46^. The radiochemical purity of [^131/125^I]FIAU was >95%, as determined by thin layer chromatography on a silica gel-coated aluminum sheet using an ethyl acetate-ethanol mixture (90:10, v/v) as the mobile phase. A Siemens E.CAM dual-head γ-camera equipped with a custom-made 1-mm pinhole collimator was used for the animal γ-imaging. To block thyroid uptake of radioiodine, mice were intraperitoneally injected with sodium iodine saline (0.9% sodium iodine in normal saline, 1 ml/animal) 15 min prior to injection of 11.1 MBq [^131/125^I]FIAU. Mice were scanned one week after [^131^I]FIAU injection to ensure clearance of urinal radioactivity. Under gas anesthesia, the animals were laid in the prone position 4 cm away from the pinhole center to provide full coverage of the body. Thirty-minute static images were collected for each animal using a 128 × 128 matrix size and zoom 2. For [^125^I]FIAU imaging, mice were scanned 2 h after probe injection by using a PET/SPECT/CT trimodality imager (GAMMA MEDICA-IDEAS, FLEX Triumph) with CT imaged at 70 kVp.

### Immunohistochemistry

The following antibodies were used in the study: anti-firefly luciferase antibody (Santa Cruz; sc-32896), anti-LHR antibody (GeneTex; GTX100008), anti-alpha smooth muscle actin (Abcam; ab7817), anti-3βHSD (GeneTex; GTX102744), and anti-FGF1 (Santa Cruz; sc-7910). Antibodies were applied to paraffin-embedded tissue sections (5 μm in thickness) followed by Alexa Fluor 488/568/647-conjugated secondary antibodies. DAPI was used to counterstain cell nuclei.

### FGF1 mRNA isolation and detection of untranslated exons

Total RNA was isolated from olfactory bulbs, forebrains, brain stems, eyes, cartilage, testes, kidneys, lungs, hearts, livers, and intestines of WT and F1B-TMIR mice by using TRIzol reagent (Invitrogen). Contamination of genomic DNA in Tg mouse RNA samples was removed by rigorous and repeated DNase I digestions, and complete removal was verified by PCR. First strand cDNA was synthesized with ReverTra Ace reverse transcriptase (Toyobo, Japan) and an oligo (dT) primer. The sequences of the PCR primers used to amplify the 405-bp eGFP region of TMIR are CCTACGGCGTGCAGTGCTTCAGC and TGCTCAGGTAGTGGTTGT. The primer sequences to amplify 413 bp of GAPDH are GTGGCAAAGTGGAGATTGTTGCC and GATGATGACCCGTTTGGCTCC. The primer sequences to amplify 270 bp of FGF1 cDNA are TACCACCGCTGCTTGCTGCC and AGGATCCTCAAGAAGTGGCC. The primer sequences to amplify 145 bp of the F1B transcript are GAGGCAGCTTCAGTCCAGGC and AGGATCCTCAAGAAGTGGCC. Quantitative PCR was performed using SYBR Green on an ABI7000 system.

## Supplementary information

Supplement information
