# Supplementary material for: A Tri-fusion Reporter Mouse Reveals Tissue-Specific FGF1B Promoter Activity in vivo
Source: Sci Rep. 2019 Jul 31;9:11143. doi: 10.1038/s41598-019-47641-3 (PMC6668445; doi:10.1038/s41598-019-47641-3)
Supplement: Supplementary file 1 — Supplement information [file 41598_2019_47641_MOESM1_ESM.pdf]

A Tri-fusion Reporter Mouse Reveals Tissue-Specific FGF1B Promoter Activity *in vivo*

Shan-Wen Liu,[1,2] Ching-Han Hsu,[2] Mei-Ru Chen,[1] Ing-Ming Chiu,[3] and Kurt M. Lin[1,4*]

[1]Institute of Biomedical Engineering and Nanomedicine, National Health Research Institutes, Zhunan Miaoli, Taiwan
[2]Department of Biomedical Engineering and Environmental Science, National Tsing-Hua University, Hsinchu, Taiwan
[3]Institute of Cellular and System Medicine, National Health Research Institutes, Zhunan Miaoli, Taiwan
[4]Department of Biomedical Imaging and Radiological Sciences, National Yang-Ming University, Taipei, Taiwan

*Correspondence author: Kurt M. Lin   klin@nhri.org.tw

Kurt M. Lin, Ph.D.
Institute of Biomedical Engineering and Nanomedicine, National Health Research Institutes, Zhunan Miaoli, Taiwan
35 Keyan Rd. Zhunan Miaoli 35053, Taiwan, R.O.C.
E-mail address: klin@nhri.org.tw
TEL: 886-37-246166 ext.37118   FAX: 886-37-586440

Supplementary information:

Supplement Figure 1

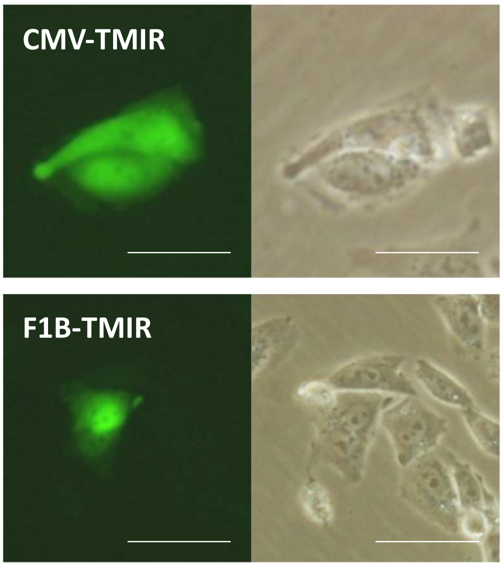

Figure S1. Fluorescence pictures of CHO-K1 cells transiently transfected with the CMV-TMIR (top) or F1B-TMIR vector (bottom). Scale bar = 100 μm.

Supplement Figure 2

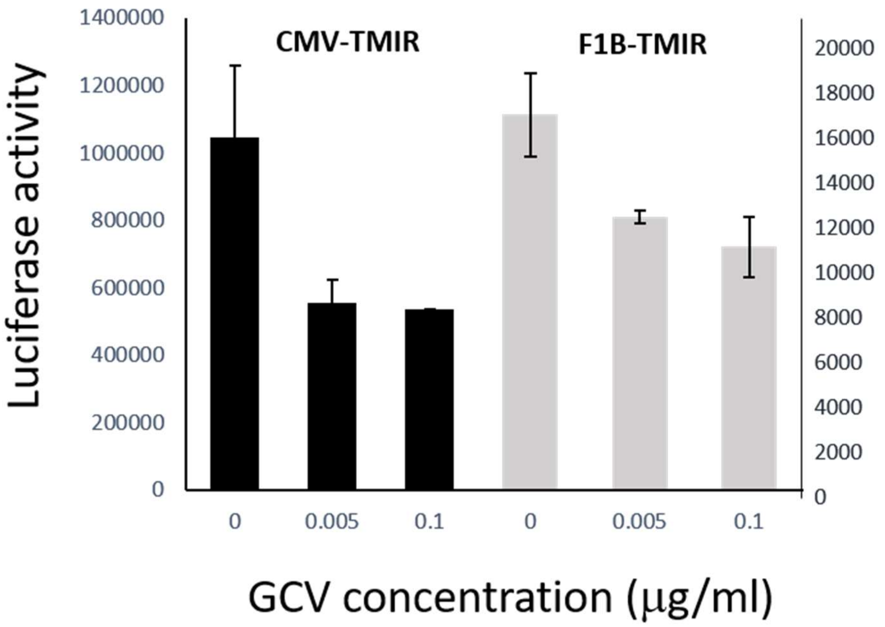

Figure S2. GCV treatment caused toxicity for the HSV1*tk*-expressing cells. GCV (0.005 μg/ml or 0.1 μg/ml) GCV reduces the luciferase activity in CMV- or F1B-TMIR vector-transfected CHO-K1 cells. The numbers shown are readings of luciferase activity assays, F1B-TMIR vector resulted in luciferase activity less than 2% of that by the CMV-TMIR vector.

Supplement Figure 3

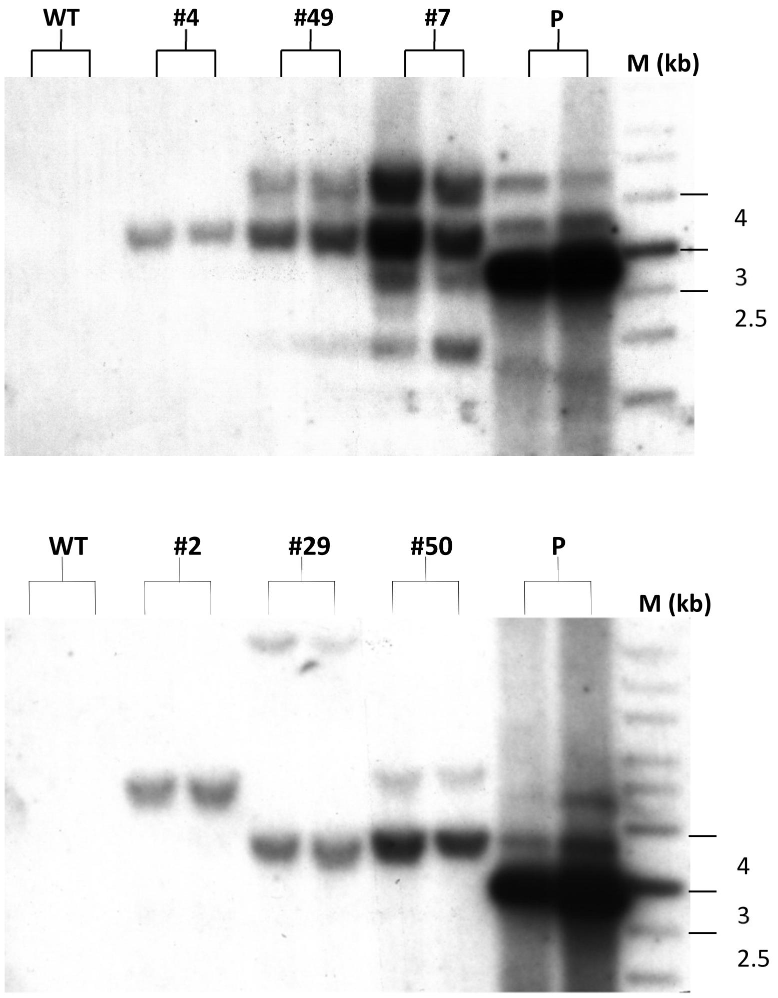

Figure S3. Southern blot of six different lines (#4, #49, #7, #2, #29, and #50) of F1B-TMIR mice. Lines #2, #7, #29, and #49 were used in this study. Marker (M), 1 and 100 pg of F1B-TMIR vector (P) as the positive control are shown.

Supplement Figure 4

A.

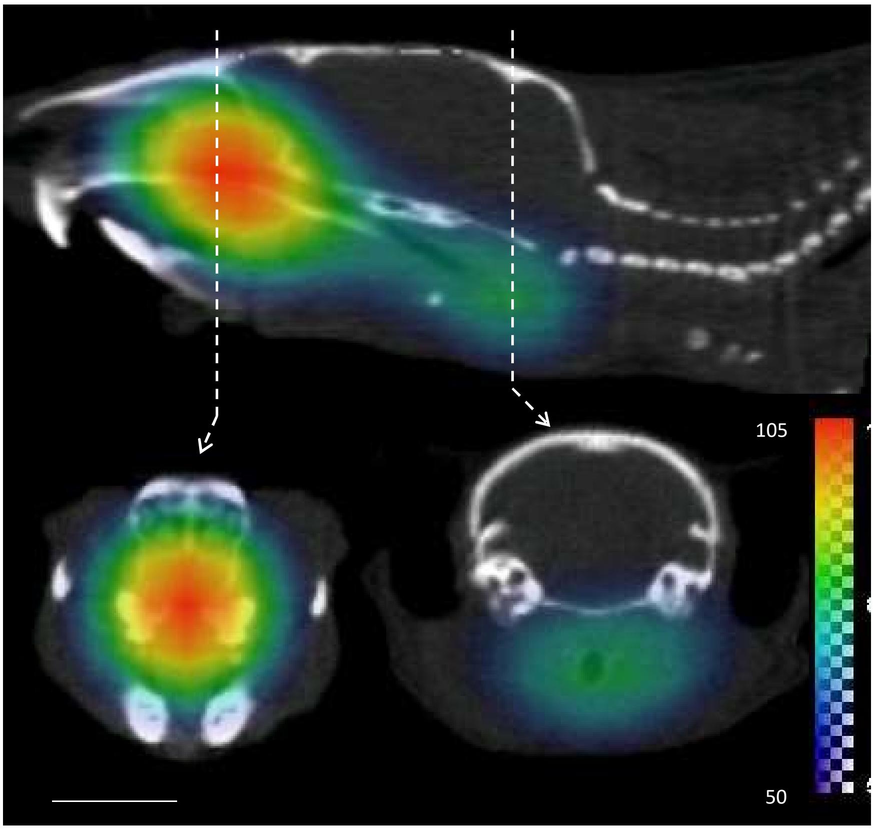

B.

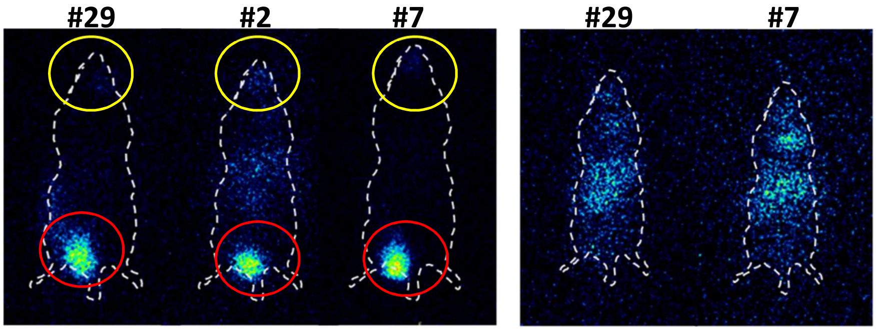

Figure S4. HSV1Δ*tk*-specific nuclear imaging. (A) [^{125}I]FIAU SPECT/CT results were in agreement with the [^{18}F]FEAU PET scan showing specific uptake at turbinate and cartilages. (B) Left, [^{131}I]FIAU γ imaging performed in F1B-TMIR mice #2, #7, and #29 one week after probe injection reveals strong uptake in the testes and low uptake in the head. Right, images of young female mice #7 and #29 for comparison, showing the absence of nonspecific probes in the abdominal region.

Supplement Figure 5

A.

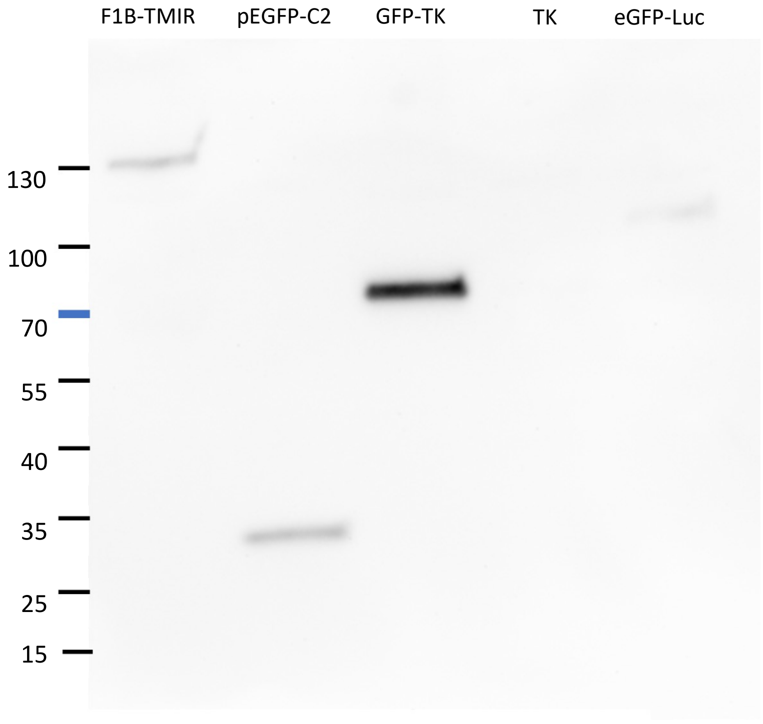

B.

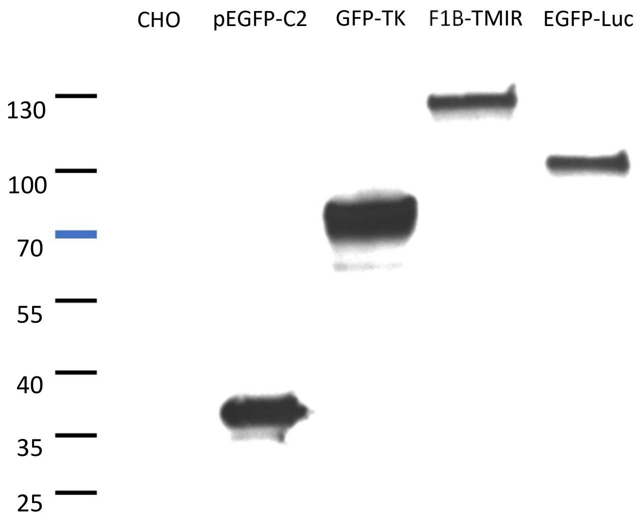

Figure S5. Western blotting of CHO-k1 cells transfected by various vectors including pEGFP-C2 vector expressing eGFP, GFP-TK vector expressing EGFP-HSV1tk fusion protein, F1B-TMIR, TK vector expressing HSV1tk, and eGFP-Luc vector expressing EGFP-luciferase fusion protein. Anti-TMIR antibody was produced by using the full-length synthetic TMIR peptide as the immunogen to raise rabbit polyclonal antibodies. (A) Anti-TMIR antibody was used in Western blotting. B) Anti-GFP antibody was used in Western blotting. The result showed anti-TMIR likely recognizing the GFP region of TMIR.

Supplement Figure 6

A.

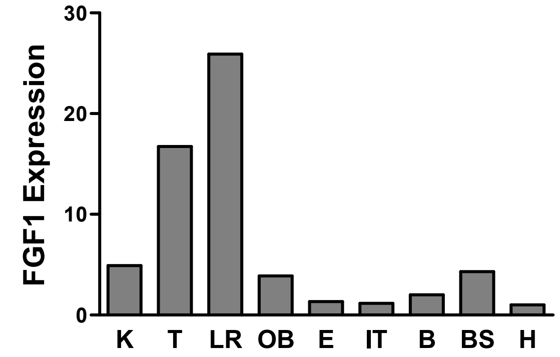

B.

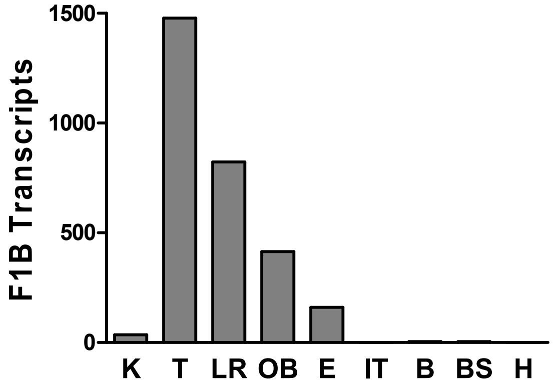

C.

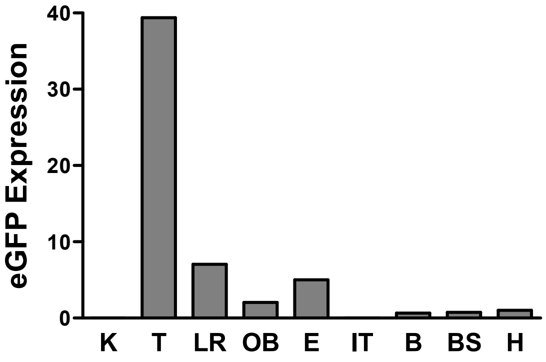

Figure S6. The expression levels of FGF1, F1B transcripts, and eGFP in various organs of F1B-TMIR Tg mice showed a correlation of TMIR expression with the activity of the mouse F1B promoter. The expression of FGF1 mRNA (A), F1B transcripts (B), eGFP (C) and GAPDH was determined by quantitative PCR using primers described in the method. The results shown were normalized to the GAPDH expression of the same organ and further normalized to the ratio of heart. Kidney (K), lung (LN), testes (T), liver (LR), olfactory bulb (OB), eyes (E), cartilage (CA), intestines (IT), forebrain (B), brain stem (BS), and heart (H).

Supplement Figure 7

A.

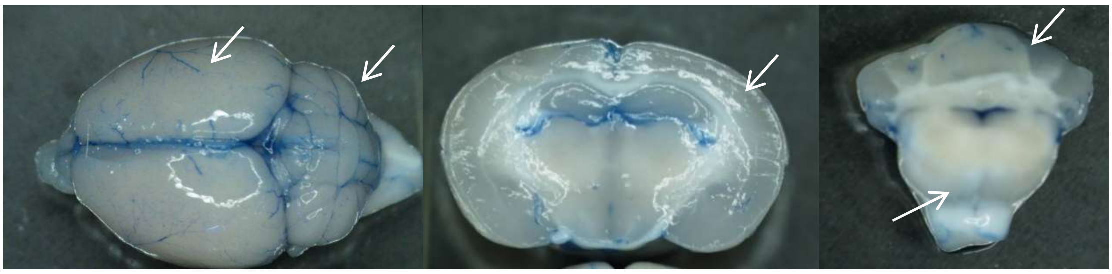

B.

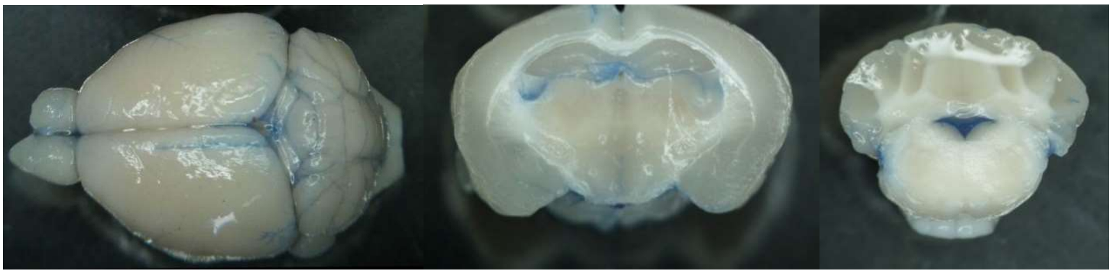

Figure S7. The effect of opening the BBB by mannitol injection. Trypan blue (0.4% in saline, 150 μl) was i.v. injected immediately following i.v. injection of 25% D-mannitol (120 μl). Ten minutes later, the mice were sacrificed and perfused with 22.5 ml of saline with 0.18% trypan blue, followed by 25 ml of 4% paraformaldehyde. After fixation, the brain was removed for observation. (A) A substantial increase in trypan blue staining in the brains of mannitol-injected mice, as shown by the darker appearance of the brain tissue relative to that (B) of control mice injected only with saline and trypan blue. The regions with the most significant increase in staining are indicated by arrows.

Supplement Figure 8

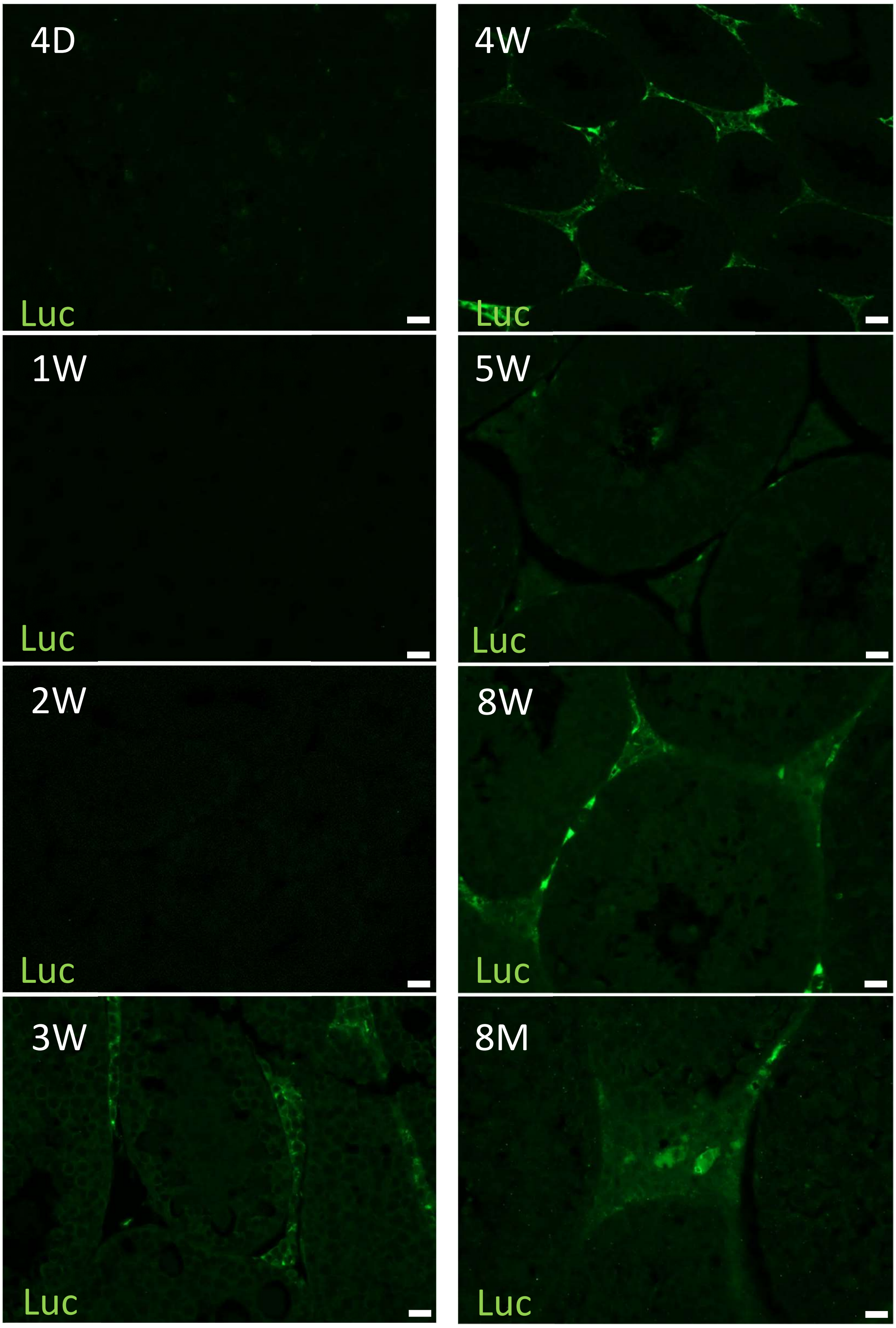

Figure S8. TMIR expression in testis Leydig cells varied during different ages. Testis samples from 4-day-old to 8-month-old were surveyed. Scale bar = 20 μm.

Supplement Figure 9

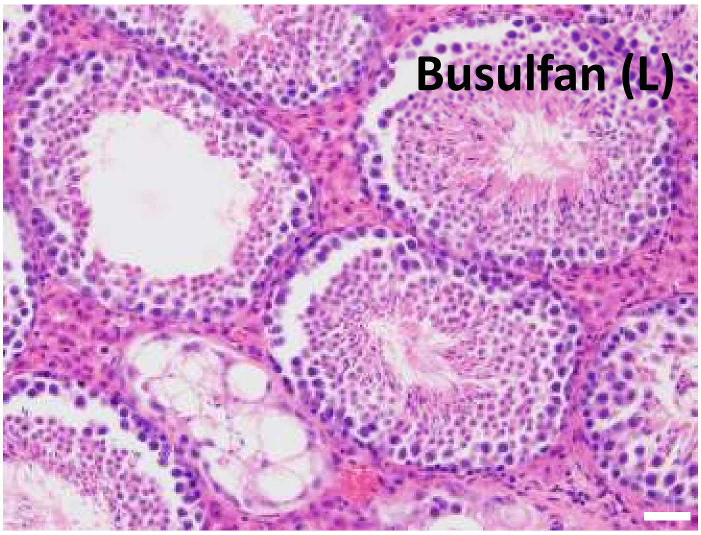

Figure S9. H&E staining of low-dose busulfan-treated testes. After a lower dose of busulfan treatment, a temporary loss of TMIR signals and the signals recovered afterward, with a slight disrupted morphology of the testis structure and only a few loss of cells of interstitial and seminiferous tubules. Most of the seminiferous tubules appeared normal. Scale bar =20 μm.

Supplement Figure 10

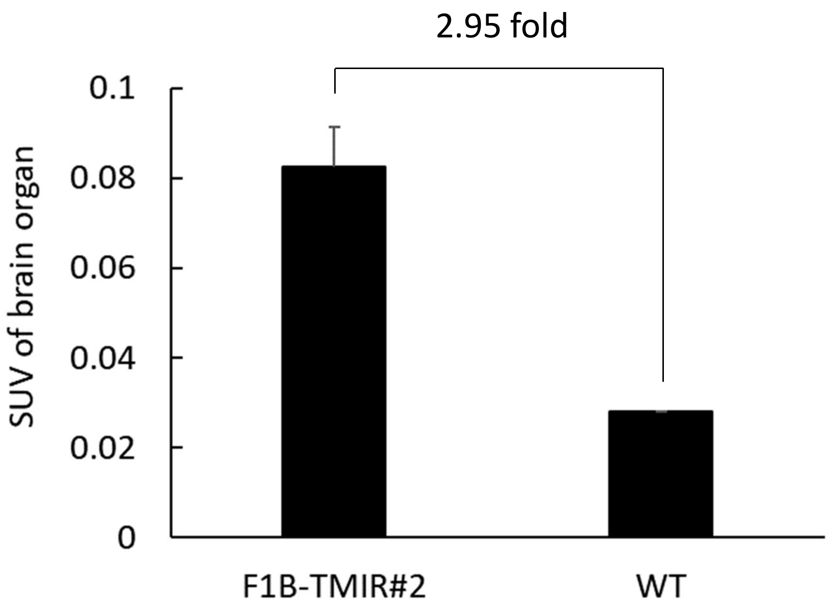

Figure S10. Standardized uptake value (SUV) of brain in F1B-TMIR and WT in [^{18}F]FEAU PET imaging. The SUV of Tg mice showed a 2.95-fold increase in SUV compared with the control group.
